# A Targeted Gene Panel for Circulating Tumor DNA Sequencing in Neuroblastoma

**DOI:** 10.3389/fonc.2020.596191

**Published:** 2020-12-14

**Authors:** Flora Cimmino, Vito Alessandro Lasorsa, Simona Vetrella, Achille Iolascon, Mario Capasso

**Affiliations:** ^1^ CEINGE Biotecnologie Avanzate, Napoli, Italy; ^2^ Dipartimento di Medicina Molecolare e Biotecnologie Mediche, Università degli Studi di Napoli Federico II, Napoli, Italy; ^3^ Department of Pediatric Oncology, Santobono-Pausilipon Children’s Hospital, Naples, Italy

**Keywords:** genetic mutation, bioinformactics analysis, next generation sequencing, neuroblastoma, liquid biopsy and circulating tumor DNA

## Abstract

**Background:**

Liquid biopsies do not reflect the complete mutation profile of the tumor but have the potential to identify actionable mutations when tumor biopsies are not available as well as variants with low allele frequency. Most retrospective studies conducted in small cohorts of pediatric cancers have illustrated that the technology yield substantial potential in neuroblastoma.

**Aim:**

The molecular landscape of neuroblastoma harbors potentially actionable genomic alterations. We aimed to study the utility of liquid biopsy to characterize the mutational landscape of primary neuroblastoma using a custom gene panel for ctDNA targeted sequencing.

**Methods:**

Targeted next-generation sequencing (NGS) was performed on ctDNA of 11 patients with primary neuroblastoma stage 4. To avoid the detection of false variants, we used UMIs (unique molecular identifiers) for the library construction, increased the sequencing depth and developed *ad hoc* bioinformatic analyses including the hard filtering of the variant calls.

**Results:**

We identified 9/11 (81.8%) patients who carry at least one pathogenic variation. The most frequently mutated genes were *KMT2C* (five cases), *NOTCH1/2* (four cases), *CREBBP* (three cases), *ARID1A/B* (three cases), *ALK* (two cases), *FGFR1* (two cases), *FAT4* (two cases) and *CARD11* (two cases).

**Conclusions:**

We developed a targeted NGS approach to identify tumor-specific alterations in ctDNA of neuroblastoma patients. Our results show the reliability of our approach to generate genomic information which can be integrated with clinical and pathological data at diagnosis.

## Introduction

Circulating tumor DNA (ctDNA), a subfraction of cell-free DNA (cfDNA), is fragmented genomic DNA poured in the blood flow and other biological fluids as the result of apoptosis and necrosis of tumor cells (1, 2). The isolation and sequencing of ctDNA from biological fluids is called Liquid Biopsy (LB). LB is a low-cost and safe non-surgical procedure to access tumor’s genetic information. It is a valid alternative for tumors that are not easy to tissue biopsy and represents a complementary tool for more accurate diagnoses. Furthermore, the ctDNA is representative of intra-tumoral and metastasis heterogeneity. The analysis of ctDNA can be useful for monitoring tumor clonal evolution and for the detection of therapy-relevant novel mutations arising during treatment (3, 4).

The development of ctDNA assays relies on the identification and quantification of somatic mutations. A variety of technical approaches have been optimized to detect diagnostic/prognostic/therapeutic markers in ctDNA with great sensitivity, including BEAMing technologies (5), panel sequencing of cancer-associated genes (6, 7), targeted amplicon sequencing (8, 9) and droplet digital PCR technologies (10). Currently, the FDA-approved qPCR test to identify Epidermal Growth Factor Receptor (EGFR) mutations, is a diagnostic test that replaces the tissue biopsy in patients with metastatic non-small cell lung cancer who would be eligible for treatment with EGFR-targeted therapy (erlotinib) (11). Nevertheless, comprehensive genomic analysis such as Whole Exome Sequencing (WES) or Whole Genome Sequencing (WGS) of ctDNA, cannot be considered in diagnostic routines because of the detection of large numbers of variants with uncertain significance. This, makes it difficult to interpret the data in a clinical setting and, in addition, often requires the profiling of a tumor tissue biopsy as a reference. However, ctDNA assays designed to target selected genes in customized panels could map tumor heterogeneity and may facilitate the identification of druggable mutations. Several commercially available ctDNA sequencing panels, designed to target specific exons or mutational hotspots, have shown their validity in clinical settings in adult cancers as in lung cancers (12).

The completely different patterns of genetic mutations between pediatric and adult cancers emphasize the need to develop specific approaches for ctDNA profiling of pediatric cancers. Recent comprehensive sequencing efforts show that only 45% of driver genes in pediatric cancers correspond to those found in adults, as demonstrated through pan-cancer studies. In children, these genes are mainly involved in biological processes belonging to epigenetic/chromatin remodelling pathways (25%). By contrast, the most relevant biological processes affected in adult cancers belong to PIK3 pathway (31%) which is altered only in the 3% of pediatric cancers (13, 14).

In the last year, feasibility of detecting, quantifying, and profiling ctDNA has been established in patients with five of the most common pediatric solid tumors: Ewing sarcoma, osteosarcoma, alveolar rhabdomyosarcoma, Wilms tumor and neuroblastoma (15, 16).

Clinical stages of neuroblastoma, according to the International Neuroblastoma Staging System (INSS), have been divided as the following: localized stages 1, 2, or 3; disseminated stage 4; and disseminated stage 4S occurring in patients younger than 1 year of age. Moreover, the International Neuroblastoma Risk Group classification system (INRG) classifies neuroblastoma patients in four categories: high-, intermediate-, low- and very low-risk (17). High-risk disease is usually diagnosed in children older than 18 months and, despite multimodal treatment, half of these patient’s relapses. *MYCN* genomic amplification is reported in 40% of high-risk neuroblastomas. It is the strongest predictor of poor prognosis and tumor progression although other chromosomal alterations are reported as poor prognostic features, namely the deletion of 1q (30%) and of the 11q (45%) and unbalanced gain of the 17q (60%) (18). Children older than 6 years present unique structural variants with 19p loss and 1q gain among those more recurrent (19). Recent whole genome sequencing analysis in a large cohort of neuroblastoma patients have identified a paucity of recurrent alterations with point mutations in *ALK* (8–10%) and in *ATRX*, being the most frequent (20, 21). The limited burden of mitochondrial DNA mutations with potential pathogenic impact have also been assessed (22). Nevertheless, a recent work has highlighted the involvement of noncoding somatic variants, located in regulatory DNA regions, in neuroblastoma development (23). Pathogenic germline variants can also contribute to neuroblastoma onset (24). Indeed, both common (25, 26) and rare germline (20, 21) variants have been found to associate with neuroblastoma development and progression.

Neuroblastoma shed high amounts of cfDNA in the blood flow depending on the tumoral burden (mean: 1.034 ng/ml of plasma; range: 13.53–26) at diagnosis and which increase with disease progression and decrease after therapy and surgical resection (27, 28). The documented high portion of ctDNA in the cfDNA fraction (mean: 60%, range: 3–99%) in high-risk diseases and metastatic cases further confirms an important shredding of ctDNA into the bloodstream (29). The majority of ctDNA studies in neuroblastoma are based on digital droplet PCR or targeted sequencing that require prior characterization of the biomarkers such as *MYCN* amplification and activating *ALK* mutations (30). The diagnostic utility of high throughput ctDNA sequencing in neuroblastoma has been published in four studies. In all cases genomic alterations detected from cfDNA were not detected from tissue biopsy. WES analysis was successfully applied to cfDNA samples at diagnosis and highlighted that ctDNA profiling performed better than primary tissue profiling in capturing tumor heterogeneity and low frequency variants. Particularly, WES analysis of primary neuroblastoma biopsies and cfDNA identified an overlap of only 41% for Single Nucleotide Variants (SNVs), whereas an overlap of 93% was observed for Copy Number Alterations (CNAs) (29). Longitudinal follow-up studies have been performed in a limited number of patients. Accumulating aberrations were found during the evolution of therapy resistant clones and some of these were potentially targetable (28, 31). Furthermore, shallow whole-genome sequencing of ctDNA to assess copy number profiles has been proposed as a valid and noninvasive genomic test alternative to the analysis of often scarce or small biopsies (32).

In this study, we report the development of a targeted Next Generation Sequencing (NGS) gene panel for ctDNA sequencing that is tailored to the genetic landscape of neuroblastoma. We believe that the ctDNA detection and sequencing, through an *ad hoc* designed gene panel, could complement or replace tissue biopsies necessary for diagnosis of neuroblastoma. The application of the suggested strategy for the diagnosis of neuroblastoma, when optimized, may improve disease stratification at diagnosis and provide useful information for therapy decisions making.

## Methods

### Patients

A total of 20 patients, with a diagnosis of neuroblastoma, were recruited at the IRCCS Istituto Giannina Gaslini and Ospedale Pediatrico Santobono-Pausilipon. Upon initial diagnosis, bone marrow biopsies and/or aspirates were obtained for microscopic examination and identification of neuroblastoma cells. Genetic abnormalities (amplification of the *MYCN* gene and deletion of the short arm of chromosome 1 [1p36]) were detected by fluorescence *in situ* hybridization. As described below, only 11 ctDNA samples qualified for deep sequencing. Of these, nine were from Stage 4 and two from Stage 2 patients. Informed consent was obtained through Research Ethics Committee of University of Naples Federico II. All experiments were performed following relevant guidelines and regulations.

### Samples Collection

Venous blood samples were collected into ethylenediaminetetraacetic acid-coated tubes and centrifuged at 1,600×*g* for 10 min. Supernatants were transferred to fresh tubes and centrifuged at 16,000×*g* for 10 min. Plasma was removed and stored at − 80 °C until DNA extraction.

### Development of Cancer Gene Sequencing Panel

The genes included in the NGS panel were selected according to these following criteria: first, we selected genes with high relevance of mutations in neuroblastoma, as reported in the Catalogue of Somatic Mutations in Cancer (COSMIC) database and PubMed; then we select genes mutated in more than two neuroblastoma samples. We included all the coding exons extended of 10 bp of flanking introns of 68 neuroblastoma-associated genes. The final length of our target was 0.5 Mb.

### NGS Library Preparation and Sequencing for Genomic Germline DNA

Genomic germline DNA was extracted from peripheral blood leukocytes using the QIAamp DNA Blood Midi Kit (Qiagen; cat #51183). A total amount of 1.0 μg genomic DNA per sample was used as input material for the DNA library preparation. Sequencing libraries were generated using Agilent SureSelect Human All Exon kit (Agilent Technologies, CA, USA) following manufacturer’s recommendations and index codes were added to each sample. Briefly, fragmentation was carried out by hydrodynamic shearing system (Covaris, Massachusetts, USA) to generate 180–280 bp fragments. Remaining overhangs were converted into blunt ends *via* exonuclease/polymerase activities and enzymes were removed. After adenylation of 3’ ends of DNA fragments, adapter oligonucleotides were ligated. DNA fragments with ligated adapter molecules on both ends were selectively enriched in a PCR reaction. After PCR reaction, library hybridize with Liquid phase with biotin labeled probe, after which streptomycin-coated magnetic beads are used to capture the exons of genes. Captured libraries were enriched in a PCR reaction to add index tags to prepare for hybridization. Products were purified using AMPure XP system (Beckman Coulter, Beverly, USA) and quantified using the Agilent high sensitivity DNA assay on the Agilent Bioanalyzer 2100 system.

### NGS Library Preparation and Sequencing for cfDNA

cfDNA isolation from 20 liquid biopsy samples was done according to the protocol (QIAamp Circulating Nucleic Acid Kit). The cfDNA quantity was assessed with dsDNA HS assay kit using Qubit Fluorometer 4.0 (Thermo Fisher Scientific). cfDNA quality was assessed with the Agilent High Sensitivity D1000 ScreenTape System (Agilent Technologies). After ctDNA quality control procedures, we excluded 9 samples due to low quality or low quantity. Therefore, eleven qualified samples could be used for sequencing. Next generation sequencing experiments on liquid biopsy samples were performed by Genomix4life S.r.l. (Baronissi, Salerno, Italy). Illumina TruSeq DNA indexed libraries were prepared from 1.7 to 10 ng of cfDNA using xGen Custom target capture Library Prep (IDT). This library preparation method incorporates unique molecular identifiers (UMIs). Libraries were quantified used Qubit Fluorometer 4.0 (Thermo Fisher Scientific) and pooled to an equimolar amount of each index-tagged sample to a final concentration of 2 nM. Pooled samples were subject to cluster generation and sequenced on NextSeq platform (Illumina) in a 2 × 150 paired-end format. The raw sequence files generated (fastq files) underwent quality control analysis with FastQC.

### Bioinformatic Pipeline

Germline DNA was sequenced by WES. Illumina paired-end reads were mapped versus the reference genome (GRCh37/hg19) using the BWA (Burrows-Wheeler Aligner; Version: 0.7.12) (33) algorithm. PCR duplicated reads were removed with Samtools (Version: 1.9) (34).

cfDNA was sequenced as described above. Sequencing reads in the FASTQ files were processed by partially modifying the IDT analysis guidelines (eu.idtdna.com) as described below.

First, we trimmed sequencing adapters. Then, we moved molecular tags (UMIs) from the read sequence to the read name (see below) with the Fastp tool (Version: 0.20.0) (35). Then, we performed read mapping by using BWA. Then, we used fgbio (Version: 0.8.1; Fulcrum genomics, http://fulcrumgenomics.github.io/fgbio/) to manipulate BAM files and extract consensus reads that were mapped again to the reference genome. In brief, we first sorted and fixed mate information. Then, we identified which reads came from the same source molecule with fgbio’s GroupReadsByUmi tool. GroupReadsByUmi was ran with the adjacency strategy (it implements the directed adjacency graph method introduced by (36) to account for sequencing errors when searching for matching UMIs. Furthermore, we required a minimum mapping quality of 30. Here, reads were aggressively filtered, only high quality read pairs were taken forward, to prevent (i) the grouping of reads that were really from different source molecules; and (ii) the building of two groups from reads that were really from the same molecule. With reads grouped by UMIs, we combined each cluster of reads to generate consensus reads using the CallMolecularConsensusReads tool of fgbio. This step generated unmapped consensus reads in BAM format that were further filtered with FilterConsensusReads tool. Here, we required that a consensus read was supported by at least two reads. Finally, we used Samtools to obtain the consensus reads in FASTQ format and run a second step of alignment with BWA. Duplicate reads marking, here was not be performed given that each read represents a unique source molecule. After the two alignment steps, we collected descriptive statistics as well as coverage metrics of targeted regions, on BAM files, by using SAMtools and Bedtools (Version: 2.29.2), respectively.

Somatic variants were called, within our targeted exons, with the VarDict software (37). VarDict is a variant calling program for SNVs, Multi Nucleotide Variants (MNVs), small Insertions and Deletions (INDELs) and complex variants. We set a minimum allele frequency of 0.01 for somatic variant calling starting from the germline BAM and consensus BAM. We, then annotated VCF files with ANNOVAR (Version: 2019-10-24).

Following the VarDict developer suggestions, we filtered variant files as follows. In brief, we kept only variants marked as “Strong Somatic” or “Likely Somatic” with a calling P value ≤0.05 (Fisher’s exact test based on read counts for variant and reference alleles in the normal and tumor samples). We also required a mean base quality of the variant in the tumor BAM > = 40. Then, we filtered out synonymous SNVs and variants with allele frequencies above 1% in European populations of 1,000 Genomes, ExAC and GnomAD. Finally, the lists of variants were filtered from those falling in ENCODE excludable regions of low mappability.

Somatic CNAs were called, within our targeted exons, with the R-Biocondutor package “CNVPanelizer” (Version: 1.14.0) installed on R (Version: 3.5.0). The tool allows reliable CNA detection in targeted sequencing applications. Its approach uses a non-parametric bootstrap subsampling of the available reference samples to estimate the distribution of read counts from targeted sequencing. We used this tool with 10,000 replicates for the bootstrap and a significance level of 0.05.

All the somatic variants that passed the filtering steps, were visually inspected by using the Integrated Genome Viewer (IGV) software.

## Results

### Patients Characteristics

Nine patients with Stage 4 and two with Stage 2 neuroblastoma were recruited as described in *Methods* (**Supplementary Table 1**). The average age at diagnosis was 36.45 months (range: 7–96). At diagnosis, three patients had *MYCN* amplification and one patient out of four tested, showed 1p36 deletion.

### Sequencing and Mapping Yield

We designed a targeted sequencing panel to cover neuroblastoma cancer driver genes and included genes with proven clinical value according to COSMIC database. The full list of neuroblastoma driver genes selected for sequencing is reported in **Supplementary Table 2**.

We have developed an experimental and bioinformatic pipeline to enable next generation sequencing and detection of somatic mutations in cfDNA. Our aim was to develop a method combining accurate sequencing technologies with rare allele amplification strategies, which could potentially be used for personalized medicine at the point of care.

The sequencing of DNA from leukocytes returned, on average 61,876 millions, of high-quality reads per sample that were mapped versus the hg19 human reference genome with a mapping rate of about 95.5%.

cfDNA sequencing yielded, on average, 45,915,594 raw reads per sample with a mean read length of 140 bp (**Supplementary Figure 1A**). Sequence duplication level was of 35.1%, on average (**Supplementary Figure 1B**). The Q20 and Q30 metrics (the percent of bases with phred-scaled quality scores greater than 20 or 30) were 89.2 and 82.1%, respectively (**Supplementary Figure 1C**). With the first mapping step, the mean depth of coverage of our target regions was about 16,752×.

After the calling of consensus reads, by exploiting the presence of molecular tags (UMIs), we obtained an average of 1,078,823 high-quality reads coming from distinct cfDNA molecules (**Supplementary Figure 1D**). Sequence duplication level was about the 0.15%, on average (**Supplementary Figure 1E**). The Q30 was 99% (**Supplementary Figure 1F**).

By aligning consensus reads, we obtained a mean mapping rate of 99.9%. The mean read depth of the targeted bases was 462.6× (**Supplementary Figure 2A**). Overall, the 81.8 and the 62% of the target bases was covered by at least 50 and 100 reads, respectively (**Supplementary Figure 2B**). The fraction of target bases covered by consensus reads was, on average, 99.3%. Indeed, only 10 exons out of 1,702 (with mean length of 112 bp range: 9–532) were not covered by consensus reads. Globally, we obtained an adequate coverage of the target genes (**Supplementary Figure 2B–C**) to perform a reliable variant calling.

### Landscape of Somatic Variants Identified in ctDNA

**Figure 1:**
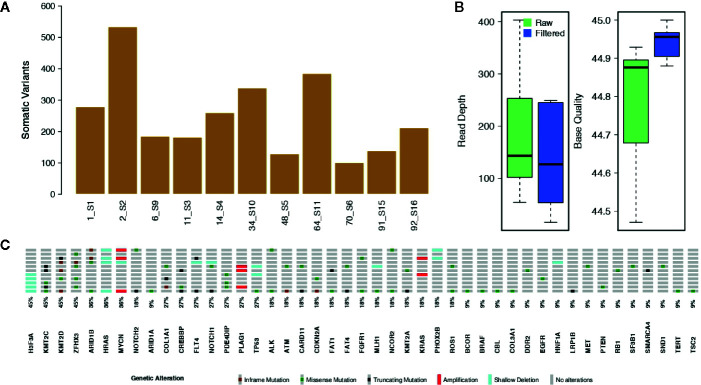
Somatic single nucleotide variants and copy number alterations. **(A)** The bar plot shows the number of raw somatic variant calls. **(B)** Box plots showing the mean Read Depth and the mean Base Quality for raw variant calls and for the filtered somatic variants. **(C)** Oncoprint reporting the somatically altered genes along with their mutation frequency.

On average, we called about 1,268 raw variants per sample. Of these, roughly the 19.3% were somatic mutations (**Figure 1A**). The mean read depth and base quality of raw somatic variant calls were 181.6 and 44.8, respectively (**Figure 1B**).

The bioinformatic analytic process was established to filter out all germline events, based on the comparison of cfDNA with the leukocyte’s DNA, and retaining only somatic alterations for further analysis. With the strong filtering described in *Methods*, we obtained a total of 101 somatic mutations, reported in **Supplementary Table 3**, (mean per sample: 9.2; range: 1–41) with allele frequencies ranging from 1.3 to 100% (mean: 12.4%; median: 6.9%). The filtered variants showed a mean read depth and base quality of 285.4 and 44.8, respectively (**Figure 1B**). Among the filtered somatic mutations, 74 were missense (73.3%), 17 were truncating (16.8%; including nine frameshifts and eight stop codon gains) and 10 (9.0%) non-frameshift INDELs (**Figure 1C**).

**Figure 2:**
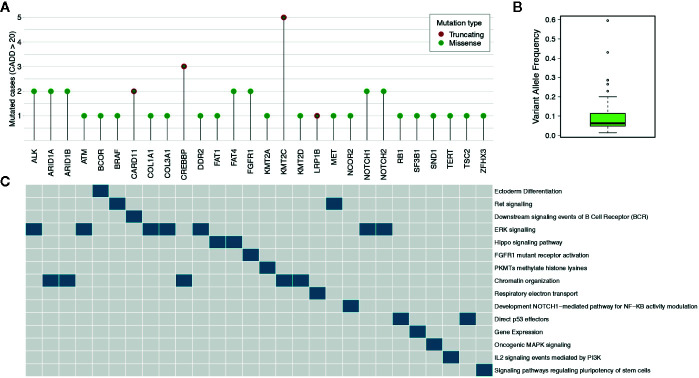
Highly pathogenic somatic mutations. **(A)** The lollipop plot shows the number of cases for genes having somatic mutations with pathogenicity scores higher than 20 as determined by CADD tool. **(B)** The box plot reports the median variant allele frequency of the selected somatic mutations. **(C)** The data matrix shows the pathways in which the mutated genes are involved (pathcards.genecards.org).

In order to identify variants with high pathogenic effects, we focused on those having a CADD (38) score above 20. We obtained a total of 51 (50.5%) filtered somatic mutations, reported in **Supplementary Table 3**, (mean per sample: 5.6, range: 1–24). Nine out of 11 cases (81.8%) harboured at least one pathogenic mutation. Among the filtered somatic mutations, 44 were missense (86.3%) and seven were stop codon gains (13.7%). Of these highly pathogenic mutations, some recurrently involved *KMT2C* (five cases), *NOTCH1/2* (four cases), *CREBBP* (three cases), *ARID1A/B* (three cases), *ALK* (two cases), *FGFR1* (two cases), *FAT4* (two cases) and *CARD11* (two cases), as depicted in **Figure 2A** and **Supplementary Figure 3**. For these mutations the median variant allele frequency was 6.2% (mean: 10.3%; range: 1.3–59.6%) (**Figure 2B**). ERK signalling and Chromatin organization were the mainly altered pathways (**Figure 2C**).

As shown in **Supplementary Table 3**, eight out of 11 patients harboured somatic variants in genes that have been previously observed in solid cancers in at least one case (as reported in the COSMIC database). Interestingly, we found the hotspot mutation F1174L in *ALK*, frequently observed in neuroblastoma tumors.

### Landscape of Somatic Copy Number Alterations Identified in ctDNA

We predicted CNAs by comparing ctDNA with its control DNA. Significant CNAs are reported in **Figure 1C** and **Supplementary Table 4**. Individual level Copy Number profiles for all the genes in our panel are in **Supplementary Figures 4–13**. We knew, by FISH assay, that three patients had the amplification of *MYCN* locus. For all of these samples we were able to confirm their *MYCN* amplification status. Furthermore, we found the heterozygous deletion of *H3F3A* in five samples. *H3F3A* mutants are drivers of tumorigenesis in pediatric cancers (39), and *H3F3A* is also a susceptibility gene in pheochromocytomas and paragangliomas (40). We also found deletions of *HRAS* in four samples and amplification of *KRAS* in two samples. These data should be treated with caution as *HRAS* is located at 11q5.5 that is a region of allelic imbalance and it can be difficult to distinguish between deletion and amplification. *KRAS* amplification has been reported in several solid cancers and is associated to lack of sensitivity to MAPK inhibitors (41). In two out of eleven samples we detected deletions of *PHOX2B* and *TP53* and amplifications of *PLAG1*. *PHOX2B* germline mutations predispose to neuroblastoma (42) and somatic variants can also occur in sporadic neuroblastoma. Although somatic mutations of *TP53* or other pathway members are rare in primary neuroblastomas obtained at diagnosis (20), *TP53* inactivation was observed in 50% of relapsed neuroblastoma (43). *PLAG1* amplifications or rearrangements have been identified in several tumors (44, 45).

## Discussion

Cancer is the leading cause of disease-related death in children. Two thirds of all survivors have late effects related to inadequate therapeutic choices or adverse health-related outcomes. Therefore, precision medicine in pediatric oncology is required to further improve outcomes and decrease toxicity. As the field of precision oncology grows, the application of ctDNA sequencing could complement or substitute the analysis of tissue biopsies and provide the means to explore the heterogeneity and the evolution of tumors. The first randomized controlled trials for pediatric cancers are currently ongoing. They will elucidate the impact of LB and ctDNA analysis on clinical decision making (NGSkids: NCT02546453 and MICCHADO: NCT03496402).

Most retrospective studies, conducted on small sample cohorts, have illustrated the great potential of LB approach in neuroblastoma. In this study, despite the limited number of samples, we highlight the utility of LB approach to facilitate the detection of druggable genes/mutations in ctDNA of neuroblastoma stage 4 patients. This could accelerate the therapeutic choice or may provide clinicians useful disease biomarkers at diagnosis.

We identified nine out of eleven (81.8%) patients who carried at least one pathogenic variation. The high prevalence of pathogenic variants in our cohort demonstrated the utility of a targeted high-throughput sequencing analysis, which excels in terms of the breadth of disease coverage when compared to single-gene tests. Furthermore, the detection of tumor-specific mutations in diagnostic ctDNA samples confirmed the potentiality of our panel to identify druggable mutations. This non-invasive method may address patients towards a personalized therapy. Particularly, we identified ALK F1174L hot spot mutation that was not previously evaluated in tissue biopsies at diagnosis. The presence of *ALK* point mutations, occurring in 8–10% of sporadic neuroblastoma, serve as biomarker of therapeutic sensitivity to small-molecule kinase inhibitors that are currently undergoing clinical assessment in phase I and II trials.

Furthermore, detecting mutations in key cancer genes, that are not actionable per se, could also benefit patient’s management by improving diagnosis, prognosis and treatment strategies setting (see (10, 12, 15, 45, 46)). In this context, we observed mutations in *KMT2C* (5/11 cases), in *CREBBP* (3/11 cases) and in ARID1A/B (3/11 cases). All of these genes take part in chromatin remodelling complexes and are frequently mutated in cancer. *KMT2C* and *KMT2D* histone lysine methyltransferases are among the most frequently mutated genes across a variety of cancer types (46). It has been recently reported that *KMT2C* is involved in DNA repair and genomic instability. This, opens up the possibility that *KMT2C*-associated cancers may be targeted by PARP1/2 inhibitors (47). Furthermore, loss of *KMT2D* results in hyperactivation of RAS/MAPK pathways suggesting that these tumors may be treated with MAPK pathway inhibitors (48). *CREBBP* mutations often result in loss of tumor-suppressive functions and are difficult to target therapeutically. In these patients, the use of HDAC inhibitors may serve as an additional therapeutic option (49). Moreover, tumors with *ARID1A* mutations are sensitive to the treatment with EZH2 methyltransferase inhibitors (50). Interestingly, we observed *FGFR1* mutations in two out of eleven. *FGFR1* gene was found mutated in neuroblastoma both at diagnosis and at relapse (21, 51, 52). Targeting of FGFR1 signalling is currently used in adult cancers and may represent an interesting application not yet explored in neuroblastoma.

In our study, we observed a wide range of Minor Allele Frequencies (MAFs) in the lists of somatic mutations for a given sample. This can be mainly explained by clonal mutational events (29). In the setting of treatment strategies, also low MAF somatic mutations should be accounted to avoid the spread of chemo-resistant clones. Indeed, given its potential, we may suggest that our targeted sequencing panel for ctDNA analysis could be also used to monitor treatment responsiveness.

*MYCN* amplification and 1p36 deletion are genetic alterations routinely detected in diagnostic tissues and serve as important prognostic biomarkers associated with poor prognosis. We confirmed *MYCN* amplification all of the patients. We could not be able to detect 1p36 deletion in one patient because the panel is not comprehensive of genes located in this region. Given the targeted nature of our sequencing panel, we recognize that we had limited capability in detecting large chromosomal aberrations. However, our choice allowed us to detect exon-level CNAs and drastically reduced both, sequencing costs and analysis time.

Many variables do affect ctDNA concentration into the bloodstream. These include tumor size and stage, metastasis, inflammation and therapy status, among the others. In children, additional variables such as age and tumor type should be considered. Despite neuroblastoma shows higher levels of ctDNA than other pediatric cancers (16), we are aware that highly standardized protocols for LB will become necessary to ensure reliable and reproducible results in routine clinical care.

In our study, we explored the usefulness of sequencing ctDNA by the means of a targeted panel of genes involved in neuroblastoma. By allowing the detection of somatic mutations (including low MAF and druggable mutations) and somatic CNAs, this approach could improve the clinical management of pediatric cancers in a cost-effective manner. Moreover, it represents a valuable alternative tool to avoid invasive tissue biopsies.

One limitation of this study is the relatively low number of analyzed tumors even if our set of samples (n = 11) derived from a careful selection among 20 tumors. Additional ctDNA sequencing studies using larger samples sizes are needed to validate the mutation frequencies we observed.

Our aim for the next future is to implement our custom NGS panel for ctDNA analysis to improve patient stratification at diagnosis. This may also lead to better targeted and timely therapies and reduce ineffective/inappropriate choices. Furthermore, the ctDNA analysis could be useful to monitor patients for early signs of relapse or for early diagnosis in families at high risk of developing neuroblastoma. Of course, well-designed and large-scale validation studies integrated in multicenter trials will be necessary to further delineate the clinical utility and validity of the proposed gene panel for ctDNA analysis.

## Data Availability Statement

The original contributions presented in the study are publicly available. This data can be found here: https://www.ncbi.nlm.nih.gov/sra/?term=PRJNA672255.

## Ethics Statement

The studies involving human participants were reviewed and approved by University of Naples Federico II. Written informed consent to participate in this study was provided by the participants’ legal guardian/next of kin.

## Author Contributions

FC designed research, performed sample preparation, interpreted the data and drafted the manuscript. VAL performed bioinformatic analysis, interpreted the data and drafted the manuscript. SV contributed with patient samples and clinical information. MC and AI formulated the strategy and supervised the research. All authors contributed to the article and approved the submitted version.

## Funding

This study was supported by grants from Associazione Italiana per la Ricerca sul Cancro (Grant no. 19255 to MC and Grant no 20757 to AI); Fondazione Italiana per la Lotta al Neuroblastoma (to MC); Associazione Oncologia Pediatrica e Neuroblastoma (to MC) and Fondazione Umberto Veronesi (to FC); Regione Campania “SATIN” grant 2018-2020 (to MC).

## Conflict of Interest

The authors declare that the research was conducted in the absence of any commercial or financial relationships that could be construed as a potential conflict of interest.

The reviewer AC declared a past co-authorship with several of the authors SV, VAL, MC to the handling editor.

## Supplementary Material

Supplementary Figure 1 Quality reports of sequencing and consensus reads. **(A)** | Total number of sequencing reads. **(B)** | Level (%) of duplicated sequencing reads as result of PCR cycles. **(C)** | Percentage of bases (within sequencing reads) with quality scores above 30. **(D)** | Total number of consensus reads. **(E)** | Level (%) of duplicated consensus reads. **(F)** | Percentage of bases (within consensus reads) with quality scores above 30.

Supplementary Figure 2 Results of consensus reads mapping. **(A)** | Bar plot reporting the Mean Read Depth of target regions across samples. Grey, dashed line shows the overall Mean Read Depth. **(B)** | Percentage of regions covered at specific read depths. **(C)** | The box plots report the Mean Read Depth of target genes. *MYCN*, in panel **(E)**, is reported separately given the different scale of read depths.

Supplementary Figure 3 Recurrently mutated genes. The lollipop plots show the protein-level localization of amino-acid changes in genes mutated in at least two samples.

Supplementary Figure 4-13 Per sample Copy Number profiles. The plots show the Log_2_Ratios of the comparisons of the ctDNA (as consensus reads) with the corresponding germline DNA at gene level. Sample names are reported in the top left corner of each figure. Significant CNAs are marked by a red dot.
