# Supplementary figures and images for: A Targeted Gene Panel for Circulating Tumor DNA Sequencing in Neuroblastoma
Source: Front Oncol. 2020 Dec 14;10:596191. doi: 10.3389/fonc.2020.596191 (PMC7769379; doi:10.3389/fonc.2020.596191)

**A**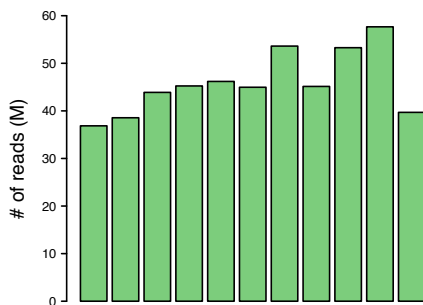**D**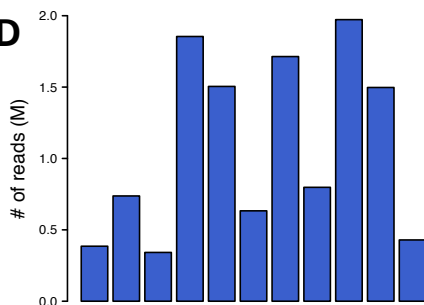**B**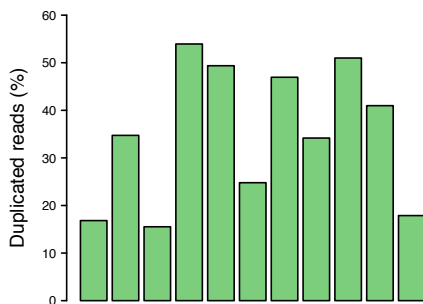**E**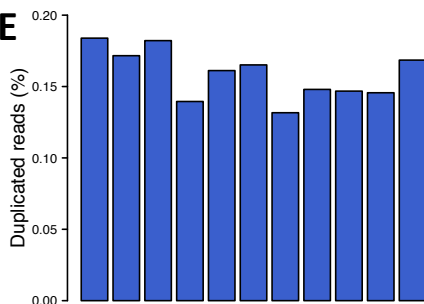**C**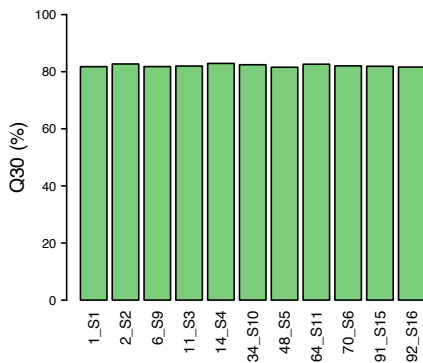**F**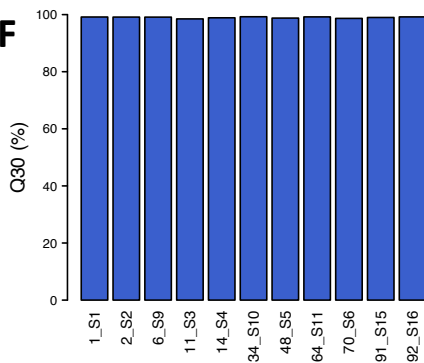

Supplement: Supplementary Figure 1 — Quality reports of sequencing and consensus reads. (A) | Total number of sequencing reads. (B) | Level (%) of duplicated sequencing reads as result of PCR cycles. (C) | Percentage of bases (within sequencing reads) with quality scores above 30. (D) | Total number of consensus reads. (E) | Level (%) of duplicated consensus reads. (F) | Percentage of bases (within consensus reads) with quality scores above 30. [file Image_1.pdf]

**A**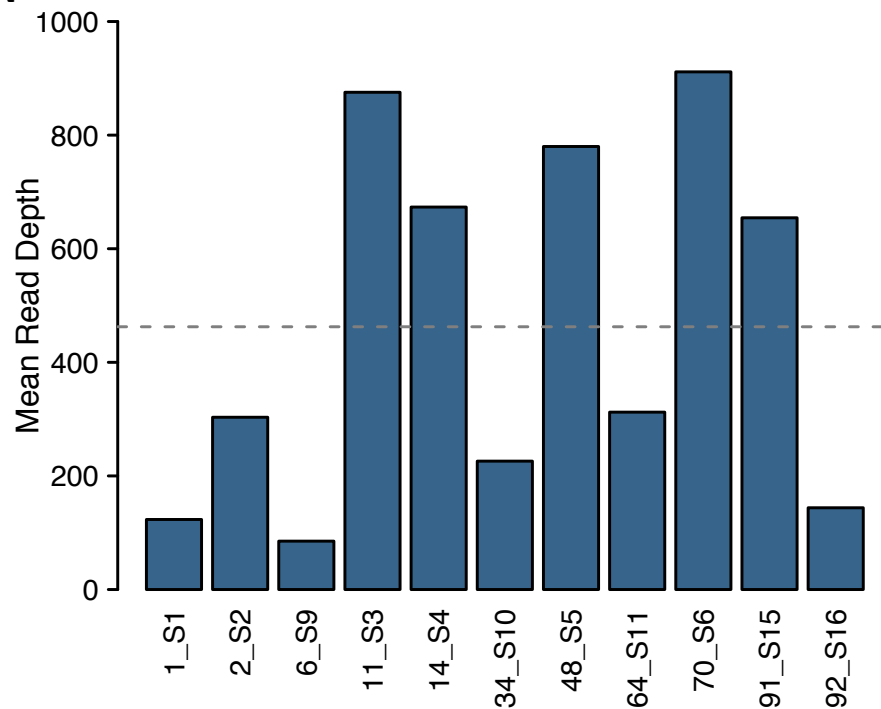**B**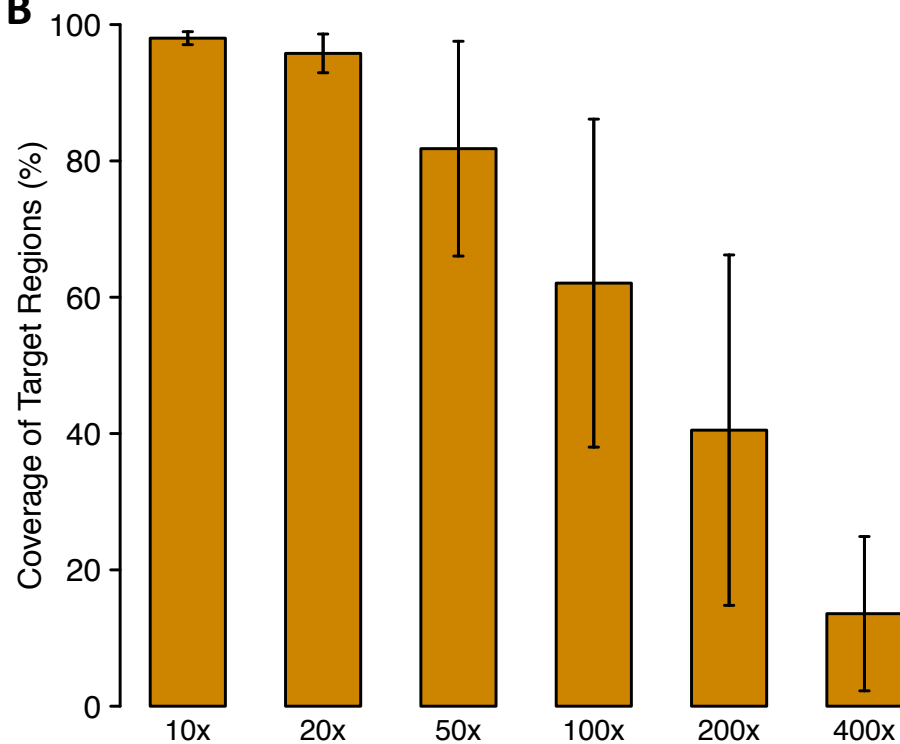**D**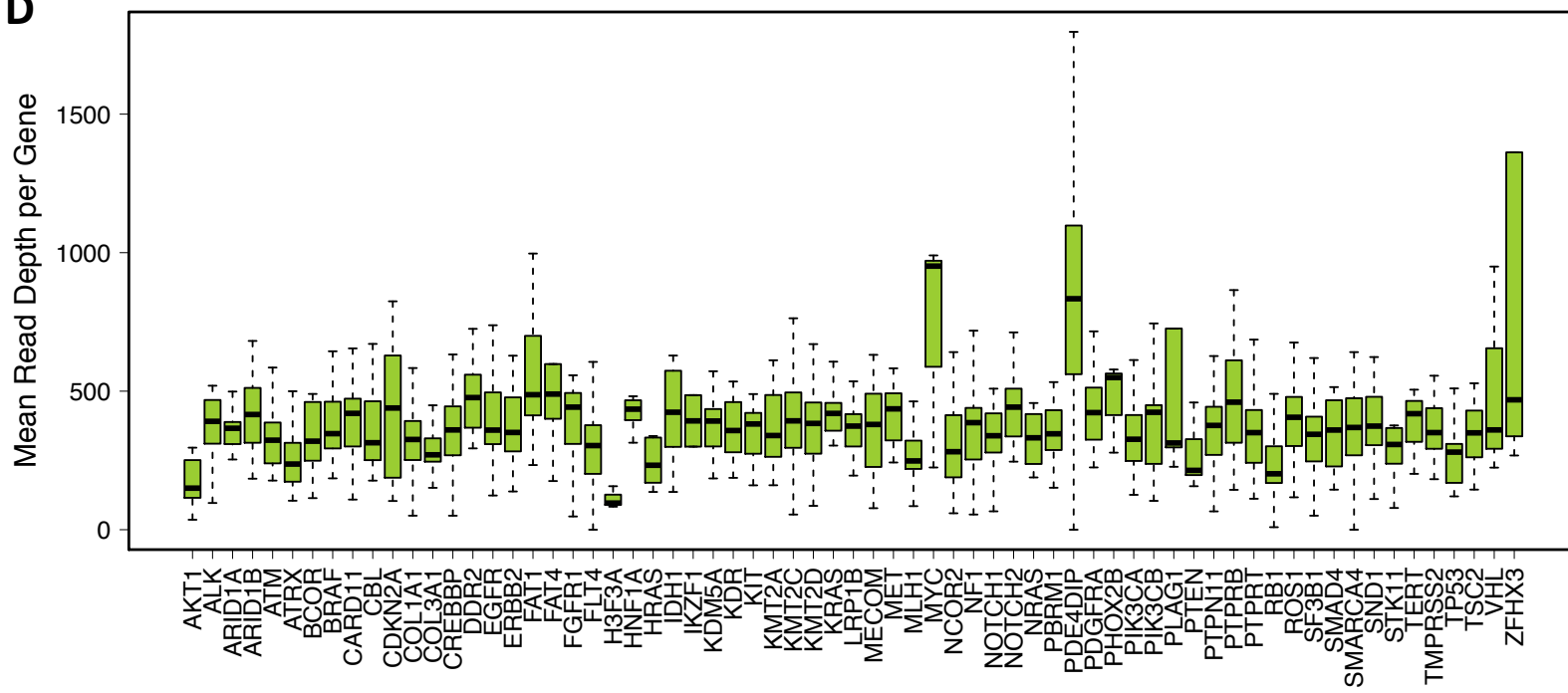**E**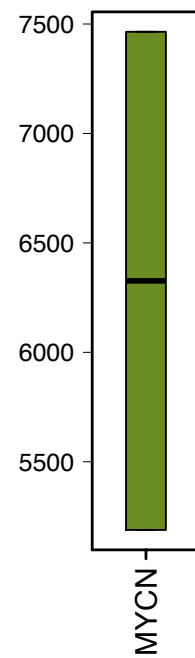

Supplement: Supplementary Figure 2 — Results of consensus reads mapping. (A) | Bar plot reporting the Mean Read Depth of target regions across samples. Grey, dashed line shows the overall Mean Read Depth. (B) | Percentage of regions covered at specific read depths. (C) | The box plots report the Mean Read Depth of target genes. MYCN, in panel (E), is reported separately given the different scale of read depths. [file Image_2.pdf]

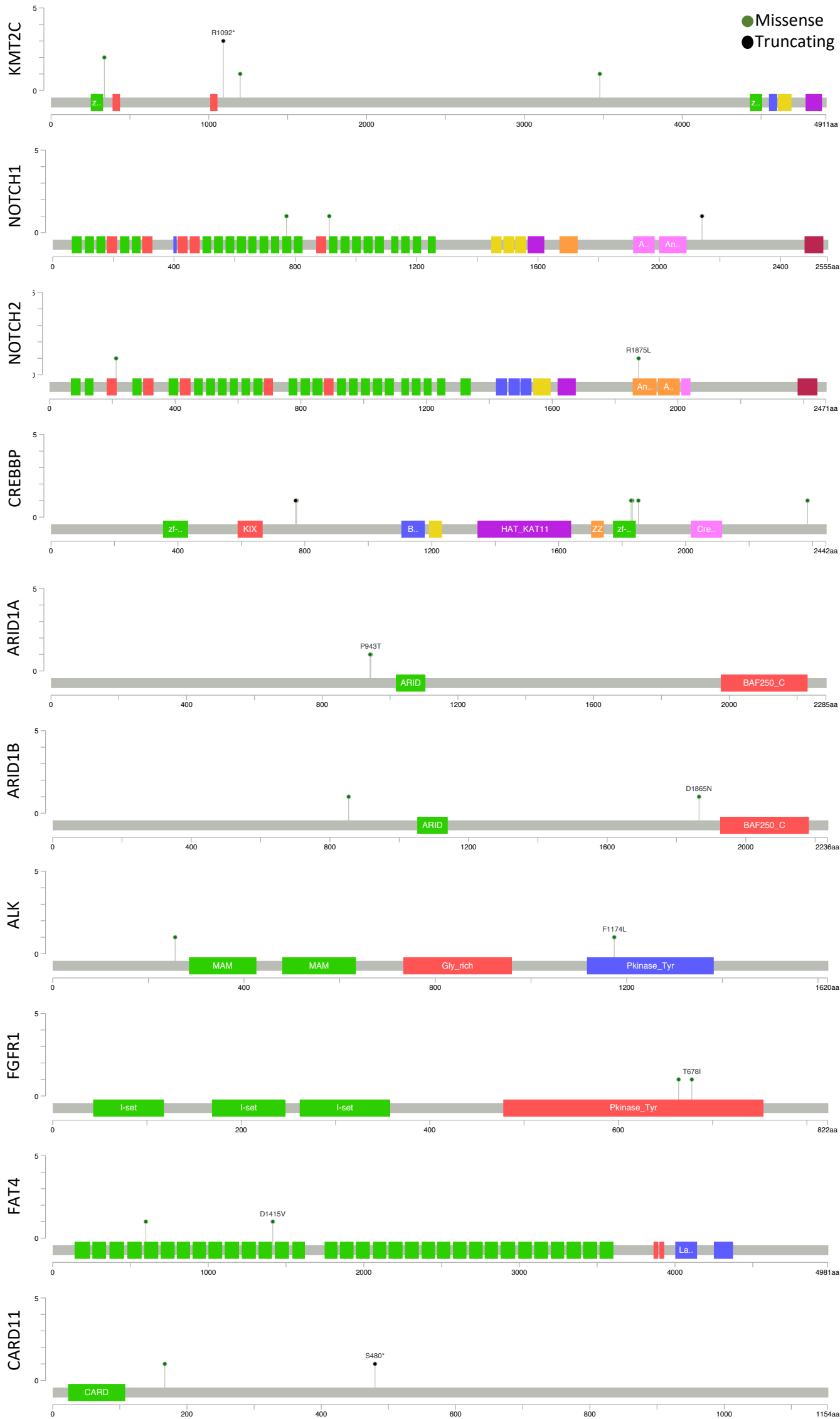

Supplement: Supplementary Figure 3 — Recurrently mutated genes. The lollipop plots show the protein-level localization of amino-acid changes in genes mutated in at least two samples. [file Image_3.pdf]

2\_S2

log2(ratios)

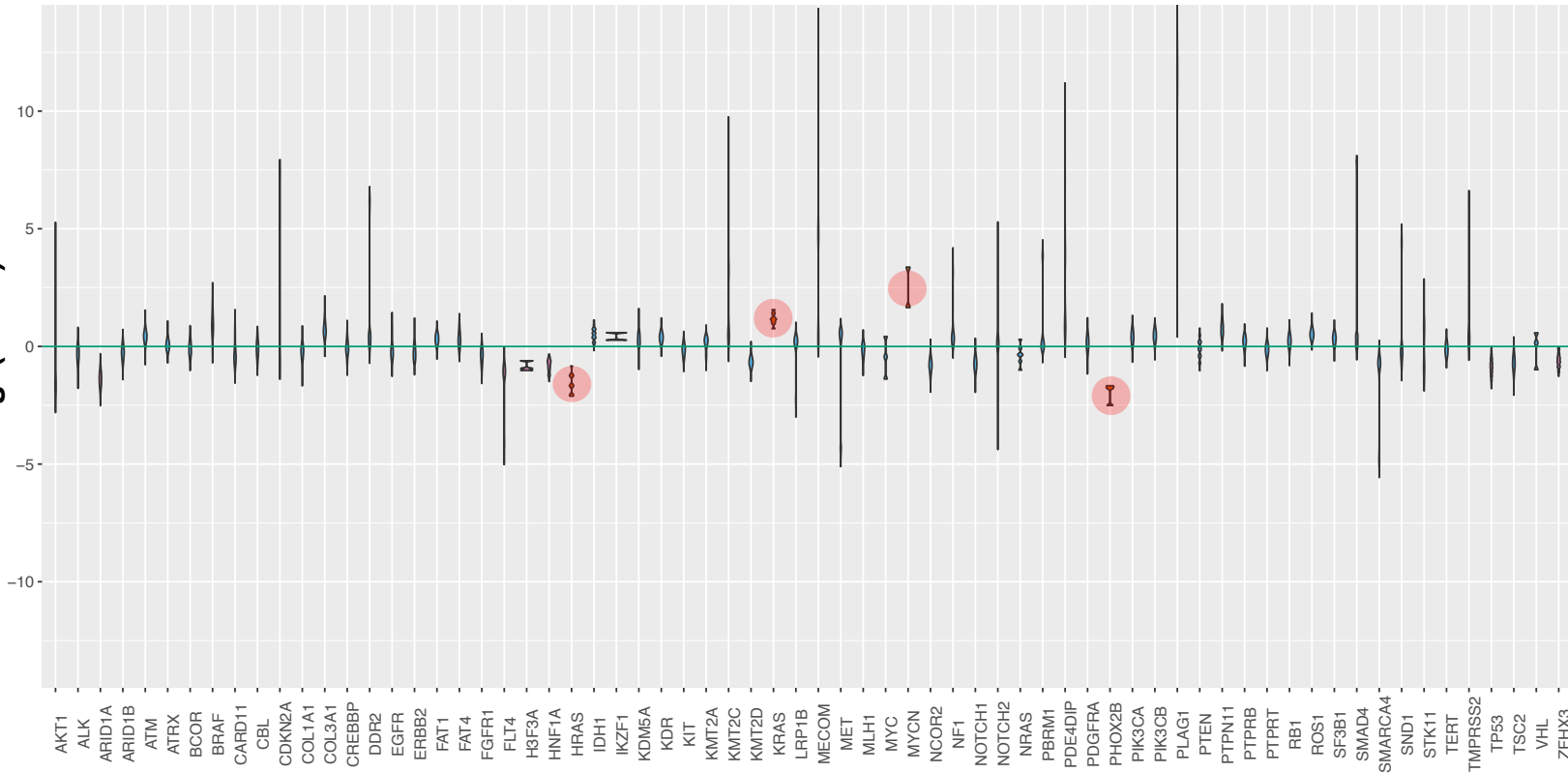

CNV Reliability

- NoChange
- NonReliableChange
- ReliableChange

Supplement: Supplementary file 5 [file Image_5.pdf]

6\_S9

log2(ratios)

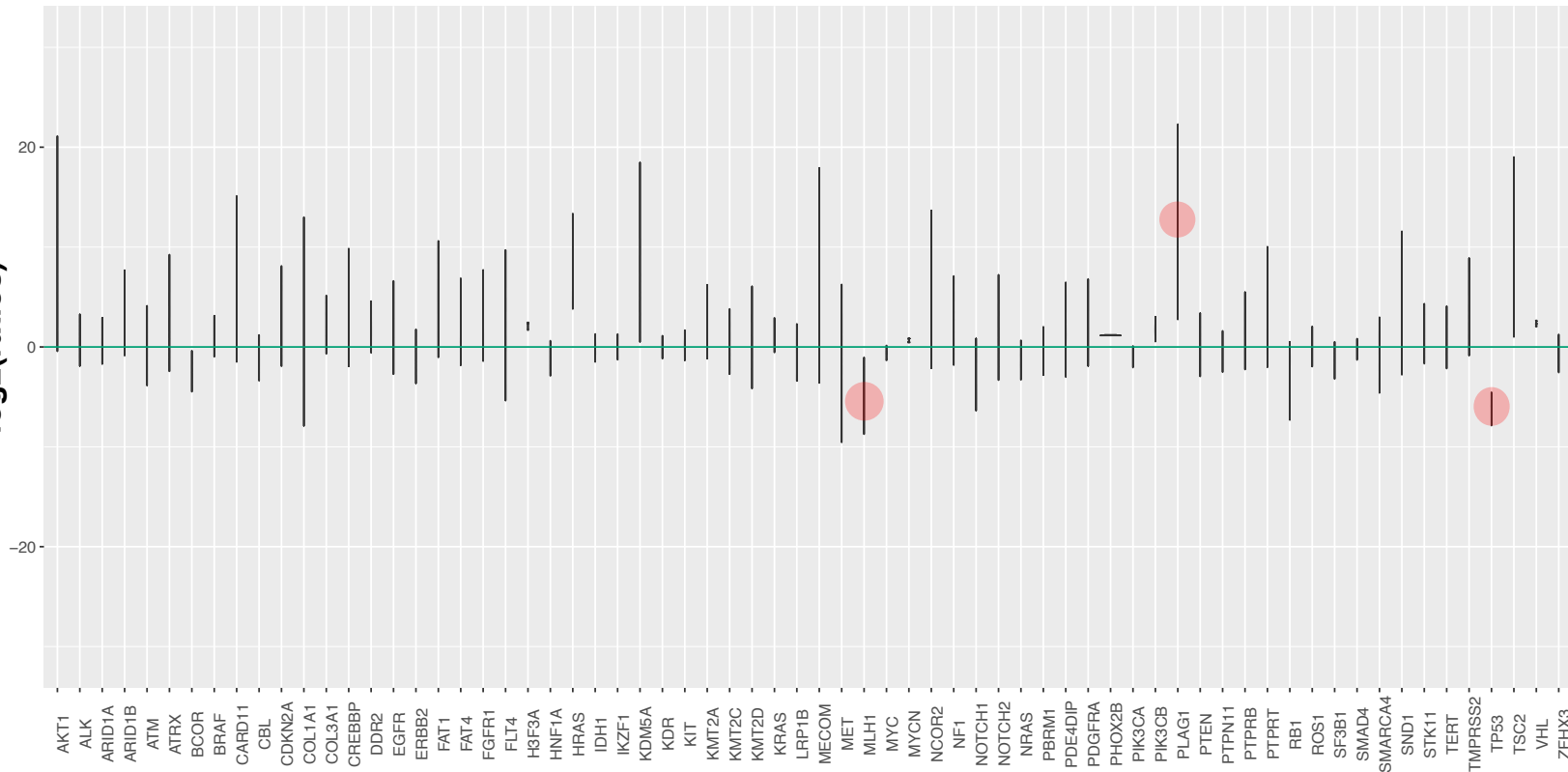

CNV Reliability

- NoChange
- NonReliableChange
- ReliableChange

Supplement: Supplementary file 6 [file Image_6.pdf]

11\_S3

log2(ratios)

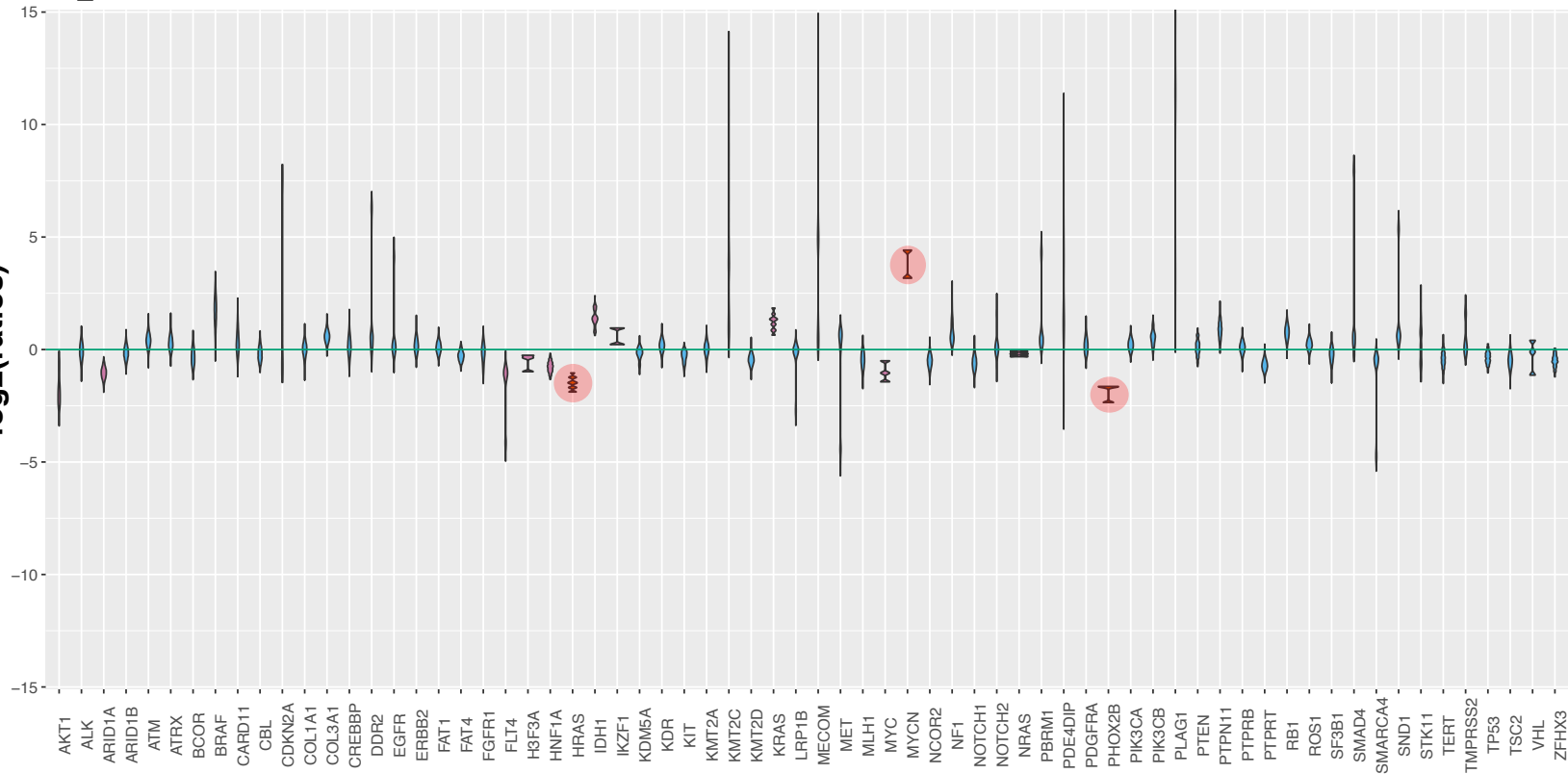

Gene Names

CNV Reliability

- NoChange
- NonReliableChange
- ReliableChange

Supplement: Supplementary file 7 [file Image_7.pdf]

14\_S4

log2(ratios)

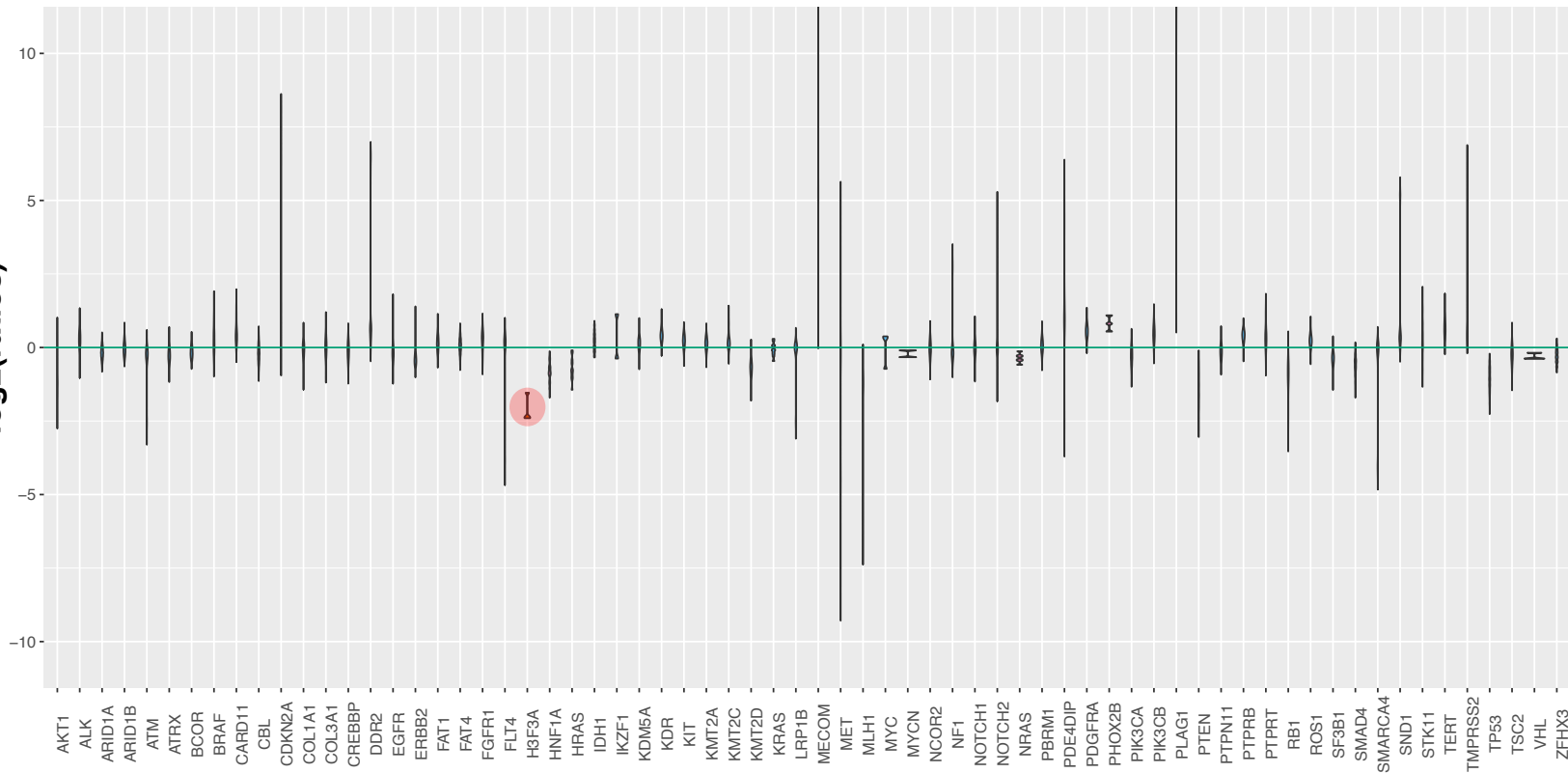

CNV Reliability

- NoChange
- NonReliableChange
- ReliableChange

Supplement: Supplementary file 8 [file Image_8.pdf]

34\_S10

 $\log_2(\text{ratios})$ 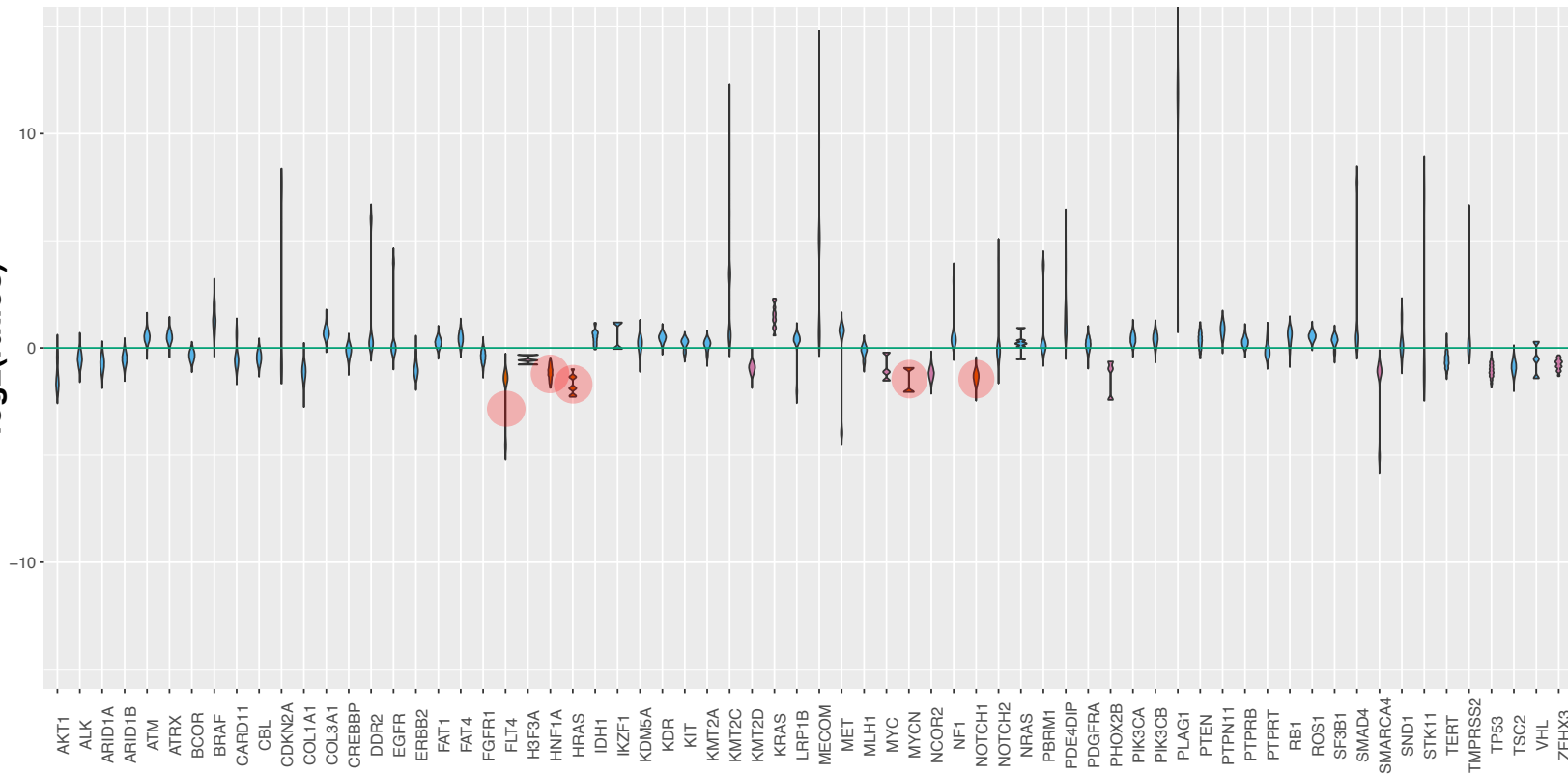

CNV Reliability

- NoChange
- NonReliableChange
- ReliableChange

Supplement: Supplementary file 9 [file Image_9.pdf]

70\_S6

 $\log_2(\text{ratios})$ 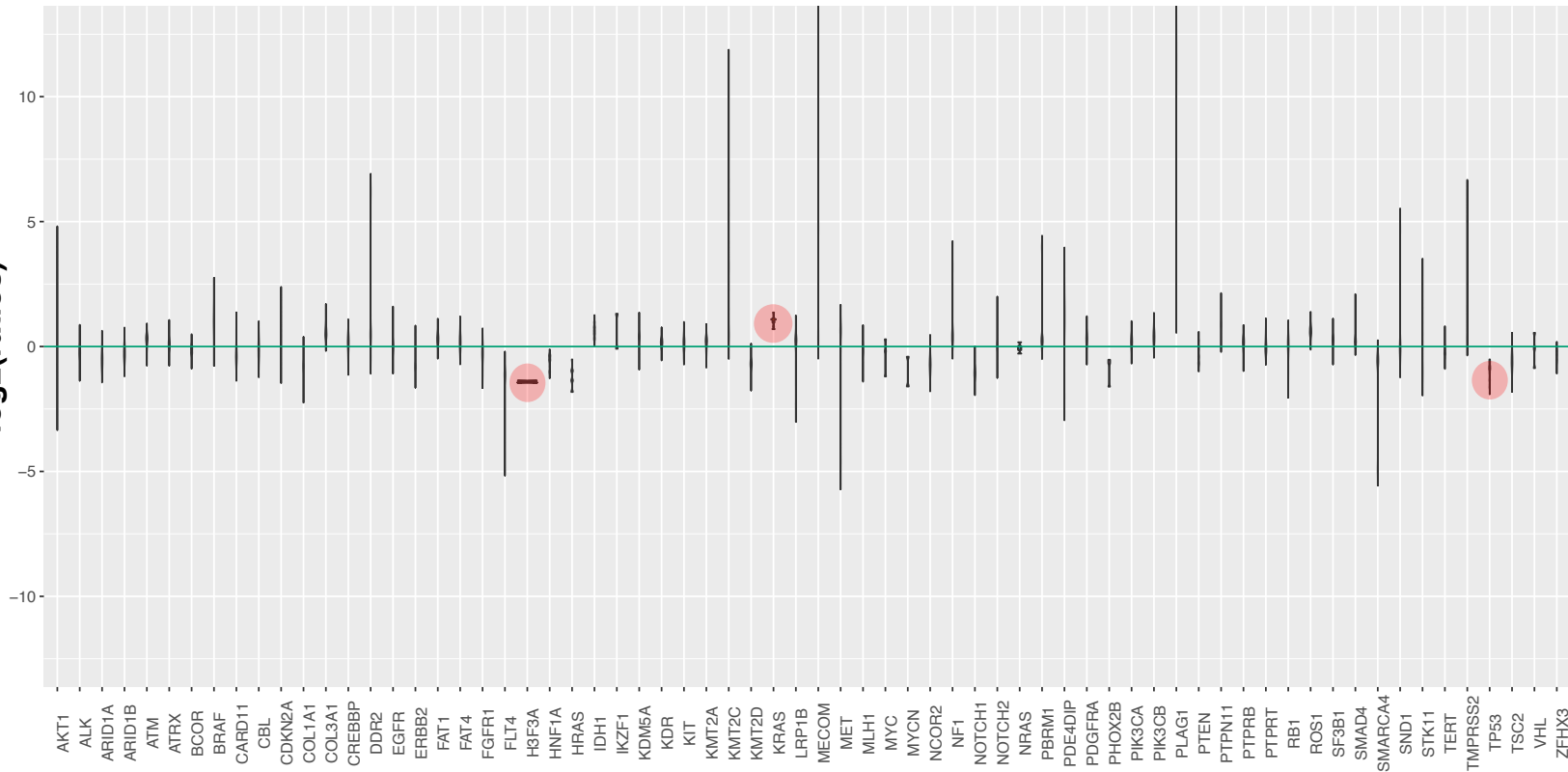

Gene Names

CNV Reliability

- NoChange
- NonReliableChange
- ReliableChange

Supplement: Supplementary file 12 [file Image_12.pdf]

92\_S16

log2(ratios)

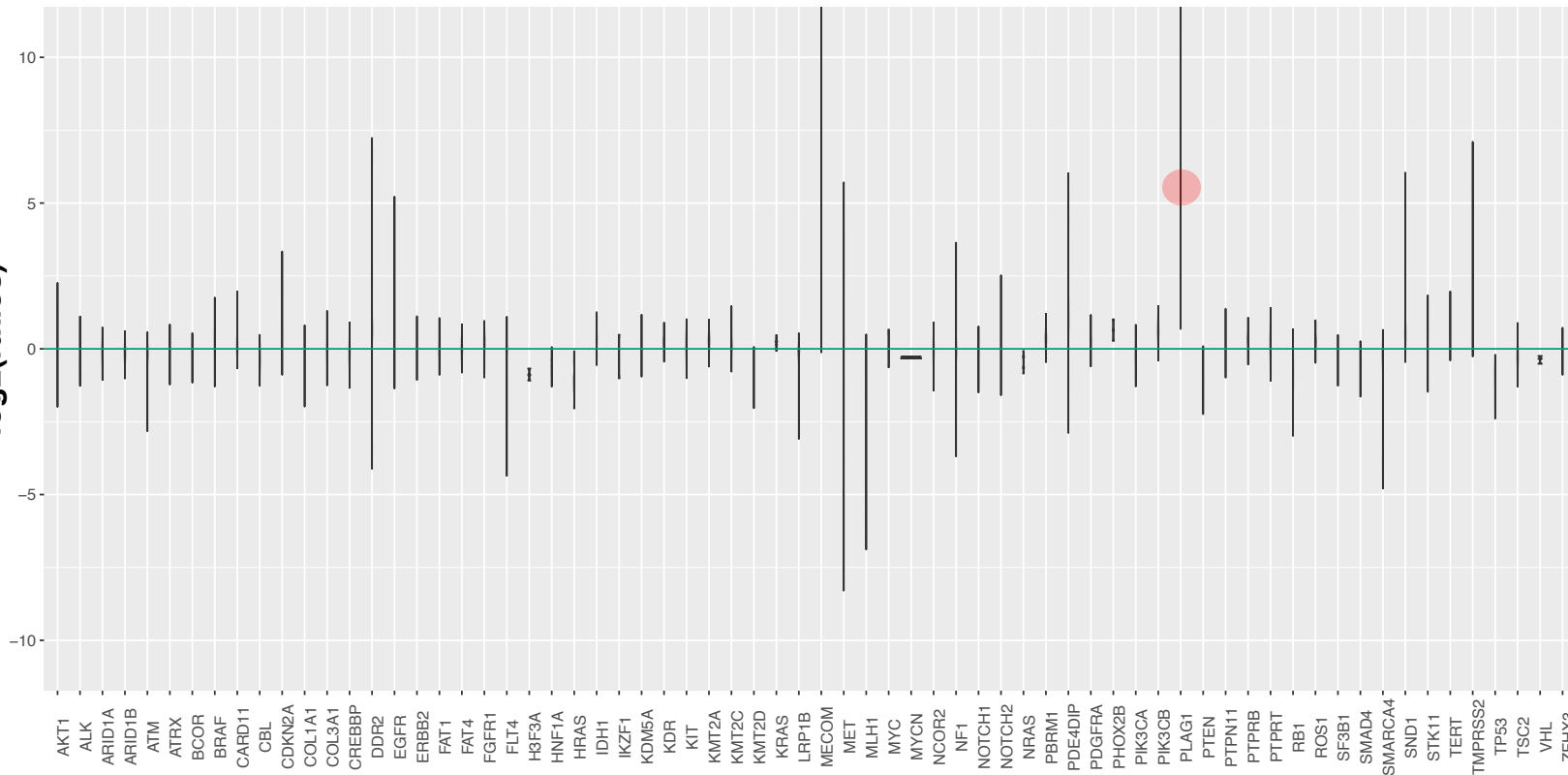

Gene Names

CNV Reliability

- NoChange
- NonReliableChange
- ReliableChange

Supplement: Supplementary file 14 [file Image_14.pdf]
