# Supplementary material for: A Targeted Gene Panel for Circulating Tumor DNA Sequencing in Neuroblastoma
Source: Front Oncol. 2020 Dec 14;10:596191. doi: 10.3389/fonc.2020.596191 (PMC7769379; doi:10.3389/fonc.2020.596191)
Supplement: Supplementary file 13 [file Image_13.pdf]

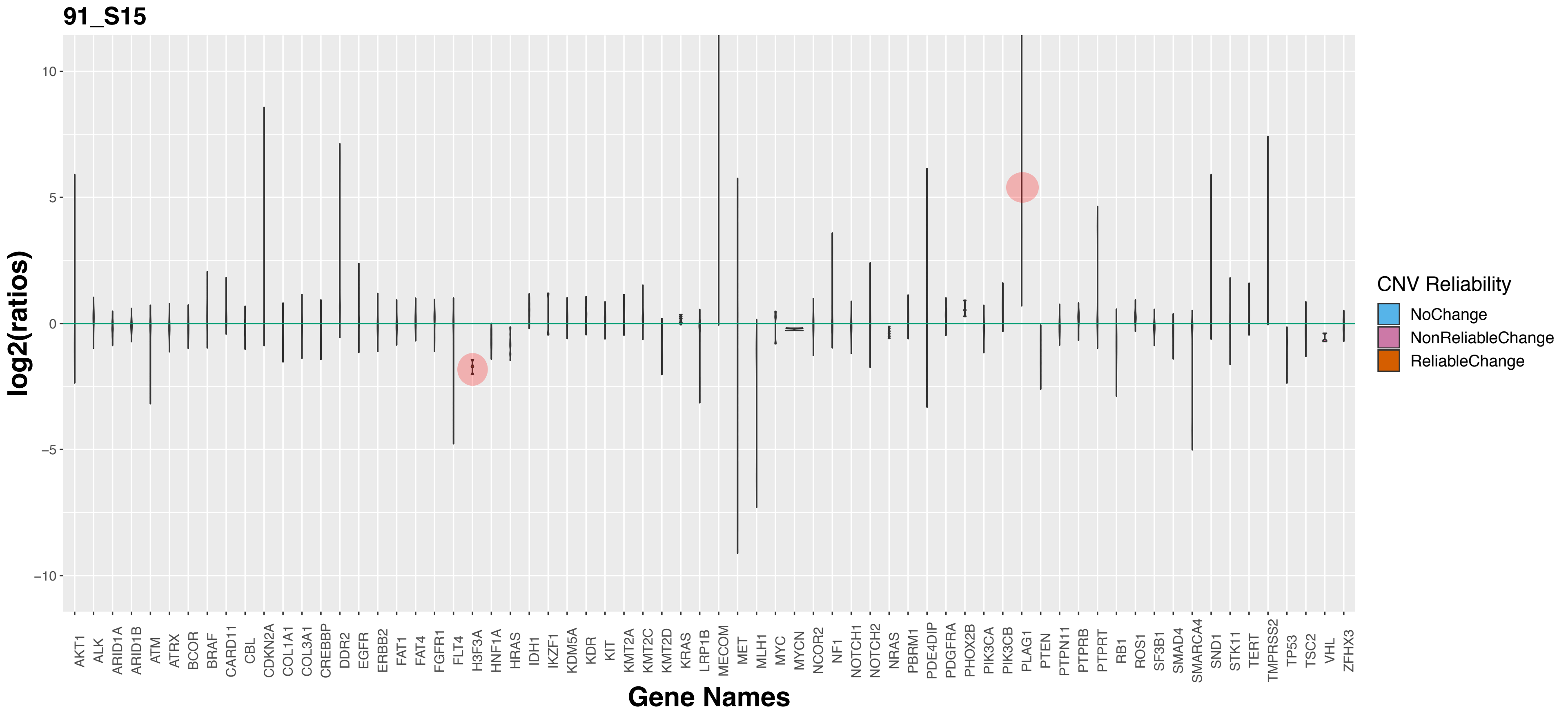
91_S15
log2(ratios)
10
5
0
−5
−10
Gene Names
AKT1
ALK
ARID1A
ARID1B
ATM
ATRX
BCOR
BRAF
CARD11
CBL
CDKN2A
COL1A1
COL3A1
CREBBP
DDR2
EGFR
ERBB2
FAT1
FAT4
FGFR1
FLT4
H3F3A
HNF1A
HRAS
IDH1
IKZF1
KDM5A
KDR
KIT
KMT2A
KMT2C
KMT2D
KRAS
LRP1B
MECOM
MET
MLH1
MYC
MYCN
NCOR2
NF1
NOTCH1
NOTCH2
NRAS
PBRM1
PDE4DIP
PDGFRA
PHOX2B
PIK3CA
PIK3CB
PLAG1
PTEN
PTPN11
PTPRB
PTPRT
RB1
ROS1
SF3B1
SMAD4
SMARCA4
SND1
STK11
TERT
TMPRSS2
TP53
TSC2
VHL
ZFHX3
CNV Reliability
NoChange
NonReliableChange
ReliableChange
